# Biotic Interactions in the Face of Climate Change: A Comparison of Three Modelling Approaches

**DOI:** 10.1371/journal.pone.0051472

**Published:** 2012-12-06

**Authors:** Anja Jaeschke, Torsten Bittner, Anke Jentsch, Björn Reineking, Helmut Schlumprecht, Carl Beierkuhnlein

**Affiliations:** 1 Department of Biogeography, BayCEER, University of Bayreuth, Bayreuth, Germany; 2 LUBW - Landesanstalt für Umwelt, Messungen und Naturschutz Baden-Württemberg, Karlsruhe, Germany; 3 Department of Disturbance Ecology, BayCEER, University of Bayreuth, Bayreuth, Germany; 4 Biogeographical Modelling, BayCEER, University of Bayreuth, Bayreuth, Germany; 5 Büro für ökologische Studien, Oberkonnersreuther, Bayreuth, Germany; Swedish University of Agricultural Sciences, Sweden

## Abstract

Climate change is expected to alter biotic interactions, and may lead to temporal and spatial mismatches of interacting species. Although the importance of interactions for climate change risk assessments is increasingly acknowledged in observational and experimental studies, biotic interactions are still rarely incorporated in species distribution models. We assessed the potential impacts of climate change on the obligate interaction between *Aeshna viridis* and its egg-laying plant *Stratiotes aloides* in Europe, based on an ensemble modelling technique. We compared three different approaches for incorporating biotic interactions in distribution models: (1) We separately modelled each species based on climatic information, and intersected the future range overlap (‘overlap approach’). (2) We modelled the potential future distribution of *A. viridis* with the projected occurrence probability of *S. aloides* as further predictor in addition to climate (‘explanatory variable approach’). (3) We calibrated the model of *A. viridis* in the current range of *S. aloides* and multiplied the future occurrence probabilities of both species (‘reference area approach’). Subsequently, all approaches were compared to a single species model of *A. viridis* without interactions. All approaches projected a range expansion for *A. viridis*. Model performance on test data and amount of range gain differed depending on the biotic interaction approach. All interaction approaches yielded lower range gains (up to 667% lower) than the model without interaction. Regarding the contribution of algorithm and approach to the overall uncertainty, the main part of explained variation stems from the modelling algorithm, and only a small part is attributed to the modelling approach. The comparison of the no-interaction model with the three interaction approaches emphasizes the importance of including obligate biotic interactions in projective species distribution modelling. We recommend the use of the ‘reference area approach’ as this method allows a separation of the effect of climate and occurrence of host plant.

## Introduction

On-going climate change is a driving factor for species range shifts (e.g. [1]–[3]). Expected range changes are often assessed by climate envelope models, which relate species’ occurrences to environmental variables [4], [5]. Such models can be projected into the future and used to detect suitable future habitats of a species and indicate potential range changes [6]. However, the restriction to climatic variables has been criticized [7], [8] and calls for the consideration of other factors determining species distributions such as biotic interactions [9].

Climate change is expected to alter biotic interactions and thereby to influence species range shifts both directly and indirectly. Positive changes, such as an escape from parasites or predators are possible [10] allowing some species to exploit a wider range of environments providing the opportunity to spread faster and in larger numbers into new areas. On the other hand, diverging influences on interacting species, such as a range contraction of the essential species, can hinder range expansions of the dependent species into new suitable areas although climatic suitability is expected (e.g. [11]). Observations and experimental studies on interactions in times of climate change are increasingly conducted (e.g. [12], [13]). However, methods to integrate interactions in species distribution modelling are still rarely implemented so far (but see [9], [11]), and no comprehensive analysis on how to best represent biotic interactions in species distribution models has been conducted.

Here, we analysed the interaction between a dragonfly, the green hawker (*Aeshna viridis* Eversmann, 1836), which is protected in the European Union under the EU Habitats Directive, Annex IV, and its egg-laying plant water soldier (*Stratiotes aloides* L.). In Europe, water soldier is nearly the only egg-laying plant of *A. viridis*, whereas this plant plays no role for reproduction in the Asian populations of the dragonfly. The restriction to *S. aloides* in Europe is advantageous for the dragonfly larvae as the spiny leaves of the plant provide shelter against fish predation [14]. Additionally, intra-guild predation and interference competition against other dragonfly larvae is reduced [15]. *S. aloides* has declined during the last decades in Europe, mainly as a consequence of eutrophication, light competition, and multiple environmental stressors resulting from water pollution [16]. With the decrease of the egg-laying plant, the dragonfly has disappeared from large parts of its European distribution and is at present highly endangered in Europe and listed in the Red Data Books of e.g. The Netherlands, Germany and Finland.

Based on the current European distribution of both species bioclimatic envelope models were developed. We applied three different approaches to consider the species’ obligate biotic interaction. First, we applied an approach that intersects the projected future distributions of both species (‘overlap approach’). Second, we used the current and future projected occurrence probabilities of *S. aloides* as additional explanatory variable for the occurrence of *A. viridis* (‘explanatory variable approach’) (similar to [9]). As third approach we restricted the climatic reference area for *A. viridis* to where the egg-laying plant is currently present (‘reference area approach’) (similar to [11]). We hypothesized that these three approaches differ considerably in their performance and in the projected extent of range change from the model without interaction and among each other. In particular, we expected a higher model performance and a lesser range change with the consideration of biotic interactions. In addition, our a priori expectation was that spatial mismatches between the dragonfly and its egg-laying plant might occur in the future.

## Materials and Methods

### Species

**Figure 1:**
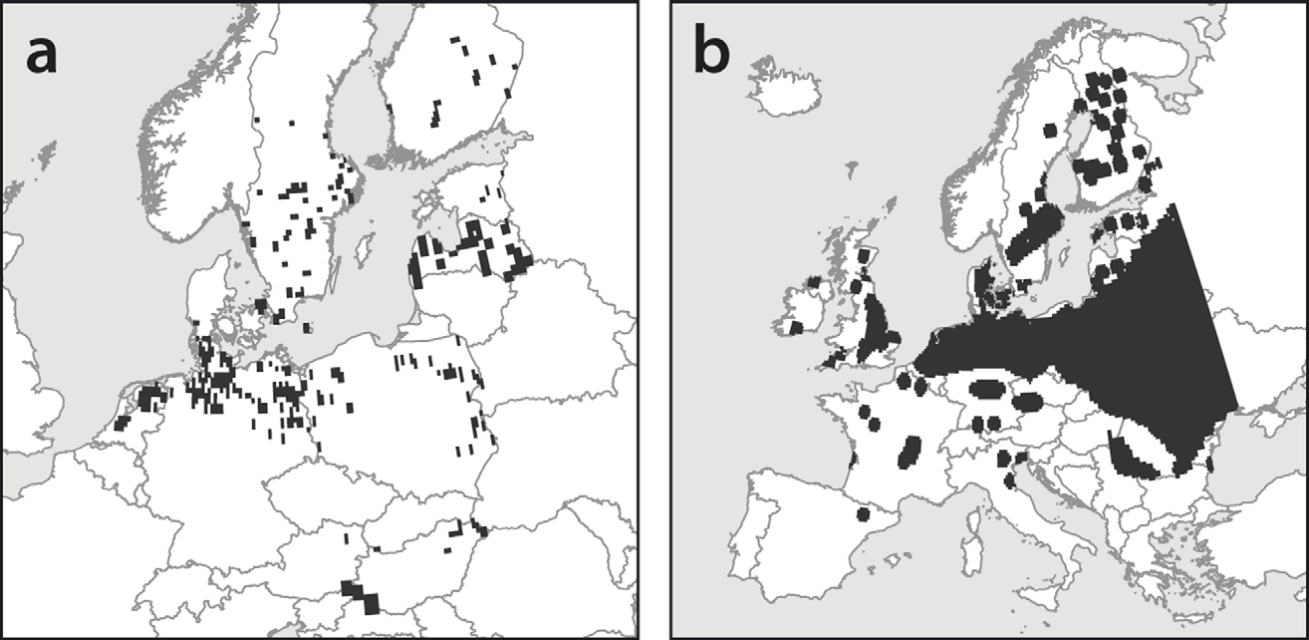
Current distribution of A) *Aeshna viridis* and B) *Stratiotes aloides* in Europe [21], [22].

The dragonfly *A. viridis* inhabits marshlands, ditches and lakes with sizeable masses of *S. aloides* in the Continental, Atlantic and Boreal biogeographical region of Europe (Figure 1A). Due to its habitat specialisation, this species is scarce and under threat in much of its European range. *A. viridis* is listed in Annex IV of the European Union Habitats Directive and therefore EU-wide protected, but is also protected by national law or under special conservation concern. Flight season is from late June onwards to early October. The species is most abundant in August [17].

The water plant *S. aloides* inhabits standing or slow-flowing, meso-eutrophic waters [18] in the same biogeographical regions as *A. viridis*, with small outposts in the Mediterranean region (Figure 1B). It exists in the shallow parts of the littoral zone as an emerged form and in deeper parts as a submerged form. During the vegetation cycle translocations of individuals between water bottom and surface occurs [14]. *S. aloides* can be used as an indicator of valuable habitat in terms of high macro-arthropod diversity and species richness [19], [20], and the occurrence of *A. viridis* further increases the conservation value of these plant populations [19].

### Species and Climate Data

Information on the current distribution of *A. viridis* was retrieved from the EIONET (European Environment Information and Observation Network) Central Data Repository server [21]. The data stem from the European reporting due in 2007 pursuant to Article 17 of the Habitats Directive. They are available for 25 EU countries in different spatial resolutions. The distribution of *S. aloides* was scanned from a map in the Atlas of North European vascular plants: north of the tropic of cancer [22] and geo-referenced in a Geographic Information System (ArcGIS 9.3.1) integrating the distribution data in our 10′ (arcminutes) grid. Distribution data of both species were provided as presence-absence data with 9932 presence points for *S. aloides* and 658 presence points for *A. viridis*. These distribution data were reported by the member states of the European Union Habitats Directive in 2007 (25 EU members). Each member has an obligation to report the distribution and state of species and habitat types protected by the Habitats Directive every six years.

Current and potential future European climate was quantified on a 10′ (arcminutes) grid from interpolated observed and future simulated climate data [23]. Future projections were based on the intermediate BAMBU (Business As Might Be Usual, A2) scenario [24], developed for the European project ALARM [25]. The future projection is driven by the HadCM3 climate model for the time period 2021–50. The observed climate data for model calibration cover the time period 1971–2000 and were taken from the ALARM dataset. Only one climate model and one emission scenario were chosen to exemplarily illustrate the application of biotic interaction approaches, although we are aware that climate models and scenarios differ among each other and therefore influence modelling results [26], [27].

The following climatic variables were used in species distribution modelling both for the dragonfly and the egg-laying plant covering the necessary ecological conditions for survival and reproduction during the activity period of the dragonfly and the vegetation period of the plant: mean monthly precipitation during the activity period of the adult dragonfly (May–August, mm), mean monthly temperature during the activity period of the adult dragonfly (May–August, °C), precipitation sum in the vegetation period (March–September, mm), sum of equilibrium evapotranspiration in the vegetation period (March–September, mm), maximum temperature of the warmest month of the year (°C), minimum temperature of the coldest month of the year (°C). Additionally, the projected current and potential future occurrence probabilities of *S. aloides* in Europe were used as explanatory variable. The average value of the projected current occurrence probability amounts to 0.35. On a local scale the existence of suitable water bodies would be additionally relevant for the occurrence of *A. viridis*. However, on the applied spatial scale (ca. 20×20 km) together with the preference of *S. aloides* for small, nutrient-rich water bodies, such as drainage ditches [18] it can be assumed that a neglect of this would be less problematic in future projections.

### Species Distribution Modelling

We used the ensemble modelling approach of BIOMOD [28], [29] with nine different modelling algorithms (generalised linear models (GLM), generalised additive models (GAM), multivariate adaptive regression splines (MARS), classification tree analysis (CTA), flexible discriminant analysis (FDA), artificial neural networks (ANN), generalised boosted models (GBM), random forests (RF), and surface range envelope (SRE)). BIOMOD allows the calculation of an ensemble prediction of all algorithms, reducing the uncertainties arising from using only a single algorithm. It provides several methods to calculate the ensemble, such as probability mean and weighted mean. We here used the probability mean, which has been reported to provide more robust predictions than other consensus methods [30]. Additionally, BIOMOD provides an assessment of variable importance based on the extent to which model predictions change when a given variable is randomized [31].

The models were trained using observed current species distribution data and observed climate data (reference period 1971–2000). The results were internally validated with a one-time data splitting method [32], randomly partitioning the data set in 70% training and 30% test data. We used the AUC (area under the receiver operating characteristic curve) as model performance criterion to measure the overall model discrimination [33]. While the AUC has been recently criticised (e.g. [34]) it still provides an informative measure of model discriminatory performance [35]. Additionally, we provide omission (fraction of observed presences projected as absences) and commission (fraction of observed absences projected as presences) rates. The threshold for occurrence and non-occurrence projections corresponds to the prevalence of model-building data [36]. A certain threshold was selected to delineate potential future range borders for calculating the projected proportion of percentage gain and loss (e.g. [37]).

All analyses were performed with R 2.12.0 [38]. In addition to the provided R packages we used the BIOMOD package version 1.1–5 [39] and the package hier.part version 1.0–3 [40]. Spatial data were processed with ArcGIS 9.3.1.

### Biotic Interaction Approaches

**Figure 2:**
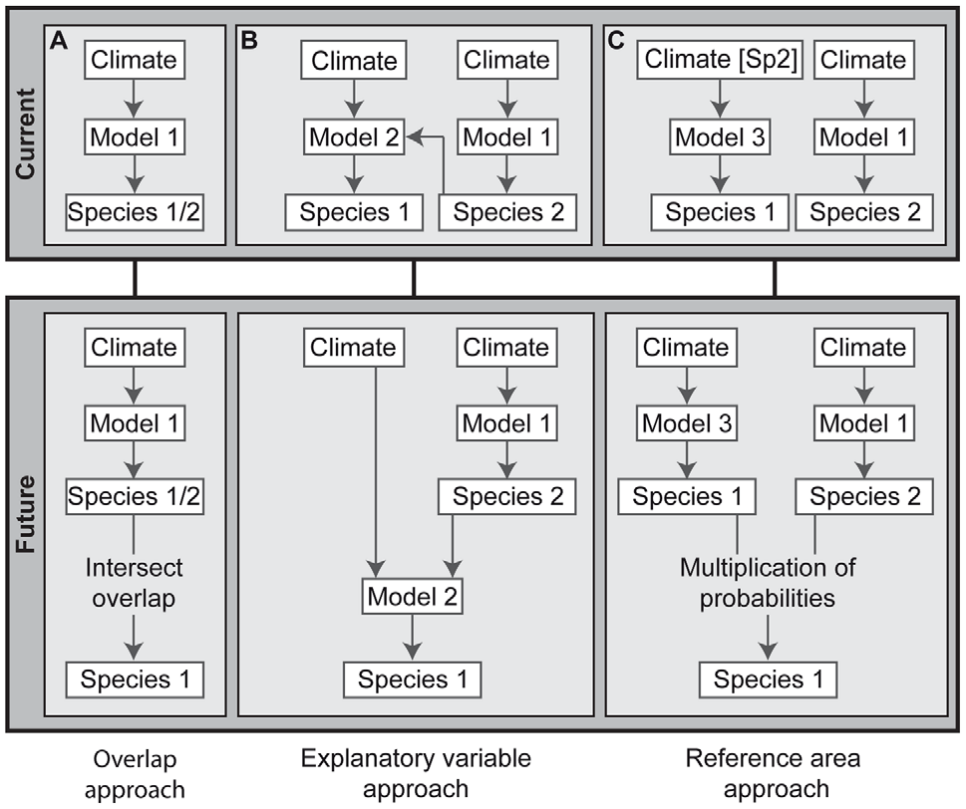
Conceptual framework of the three applied approaches for modelling biotic interactions. A) ‘Overlap approach’: modelling both species separately and intersecting the future range overlap. B) ‘Explanatory variable approach’: modelling the dependent species with the essential species as additional explanatory variable. C) ‘Reference area approach’: modelling the dependent species in the range of the essential species and multiplication of the occurrence probabilities of both species. Species 1: dependent species (here: *Aeshna viridis*), Species 2: essential species (here: *Stratiotes aloides*), Climate [Sp2]: restriction of climatic reference area of species 1 to the current distribution of species 2.

For modelling the distribution of *A. viridis*, the following three approaches were applied: (1) ‘overlap approach’, (2) ‘explanatory variable approach’, and (3) ‘reference area approach’ (Figure 2). For the ‘overlap approach’, the current and potential future distributions of *A. viridis* and *S. aloides* were modelled individually with climatic variables. The projected future occurrences of both species were intersected, retaining only those areas where both species are projected to occur mutually in the future assuming unlimited dispersal (Figure 2A). The ‘explanatory variable approach’ includes for the modelling of the dragonfly, beside the climatic variables, the modelled current and projected future occurrence probability of the egg-laying plant in Europe (Figure 2B). For the ‘reference area approach’ the distribution model of *A. viridis* was calibrated on the current occurrence of *S. aloides* and then projected on Europe. This model thus describes the conditional probability of finding *A. viridis* under particular climate conditions, given that *S. aloides* is present. To yield the unconditional occurrence probability for *A. viridis*, this conditional occurrence probability was multiplied with the modelled occurrence probability of *S. aloides* (Figure 2C).

### Comparison of Interaction Approaches

We compared the results of the three approaches according to four criteria: First, we evaluated the modelling performance with the criterion AUC on test data. Second, we analysed the spatial projections. For this purpose, we identified the two most important climatic variables determining the current distribution of *A. viridis* in Europe using the variable importance function in BIOMOD. We then plotted the projected future losses and gains of all three approaches within the range of these two variables to assess where (in terms of the variable range) the projections differ.

Third, potential non-analogue climatic conditions between current conditions and future projections in time were calculated for the ‘reference area approach’, which is particular susceptible to this phenomenon as it restricts the climate space used for model fitting of the dragonfly species to that space occupied by the egg-laying plant. Non-analogue climate demands caution in the interpretation of the results [41]. Potential non-analogue climate was determined by the Multivariate Environmental Similarity Surface (MESS) analysis [42]. The MESS analysis measures the similarity between the current observed climate used to train the model and the future projected climate for any grid cell. Negative values imply non-analogue climatic conditions.

Finally, we analysed the main source of variation in modelling results, i.e. either modelling algorithm or biotic interaction approach, using hierarchical partitioning. This method measures the contribution of each applied variable, independently and in conjunction with the other variables, to the total variance of a regression model and provides its relative importance. The nine modelling algorithms and three biotic interaction approaches resulted in 27 different future projections. These were analysed by calculating the difference between the amount of gained sites (number of projected future suitable grid cells where the species is currently absent) and the amount of lost sites (number of projected future unsuitable sites where the species is currently present) relative to the number of currently occupied sites [43]. These values were related to uncertainty factors (modelling algorithm, biotic interaction approach) using a linear model with a Gaussian error distribution.

## Results

### Projected Geographical Changes

**Figure 3:**
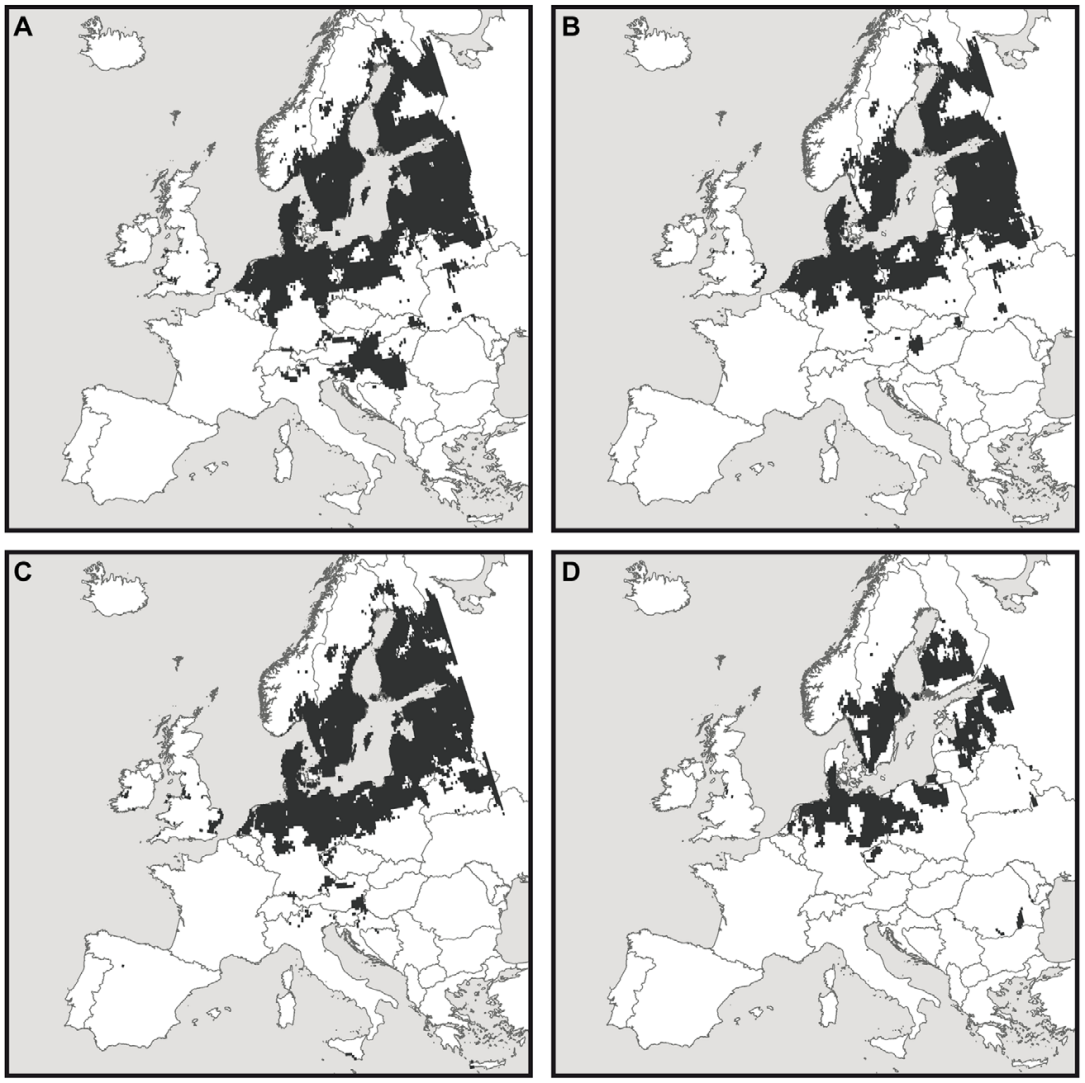
Projected potential future distributions of *Aeshna viridis* in Europe assuming unlimited dispersal. A) *A. viridis* without interaction, occurrence threshold: 0.02, AUC: 0.93. B) Overlapping area of the potential future distributions of *A. viridis* and *S. aloides*, occurrence threshold: 0.02 (*A. viridis*), 0.35 (*S. aloides*). AUC: 0.93 (*A. viridis*), 0.94 (*S. aloides*). C) Considering the modelled occurrence probability of *S. aloides* in Europe as additional explanatory variable beside climate. Occurrence threshold: 0.02. AUC: 0.92; D) Potential future distribution of *A. viridis* in Europe applying the ‘reference area approach’. The model for *A. viridis* was calibrated within the distribution area of *S. aloides*. The modelled future occurrence probabilities of both species were multiplied. Occurrence threshold: 0.05. AUC: 0.88. All modelling results are based on an ensemble modelling with nine model algorithms with the climate model HadCM3 and the emission scenario A2 for the time period 2021–50.

Modelling the future European distribution of *A. viridis* solely with climatic information leads to a projected northward range expansion of this species (Figure 3A). Overall, a substantial range gain is projected for *A. viridis* (+1069%) assuming unlimited dispersal ability.

Including the biotic interaction with *S. aloides* leads to a smaller projected range expansion, irrespective of the particular biotic interaction approach. With the ‘overlap approach’, the overlapping area of both species is projected to increase. The projected overlapping region concentrates around the Baltic Sea in the future with core areas in North Germany/Denmark, Poland, Southeast Sweden, and Estonia/Latvia/South Finland (Figure 3B). The projected gain of area amounts to 860% compared to the current range of *A. viridis* assuming unlimited dispersal.

With the ‘explanatory variable approach’, the dragonfly is projected to gain, similar to the ‘overlap approach’. The overall projected gain is, however, larger than with the ‘overlap approach’ (+984%, unlimited dispersal). The potential climatically suitable area of the dragonfly is mostly distributed around the Baltic Sea with core areas in North Germany/Denmark, Southeast Sweden and Estonia/Latvia/South Finland (Figure 3C). Some more potentially suitable areas are projected in Finland, Sweden and Poland than in the overlap approach.

The ‘reference area approach’ projected the smallest gain of suitable area in the future: The amount of the projected gain accounts for 402% with unlimited dispersal. The projected area is more fragmented and contracted around the Baltic Sea than with the other approaches (Figure 3D).

For the ‘reference area approach’, climatic similarity between calibration and projection region was determined by MESS analysis. Non-analogue climate can be identified along the Mediterranean coast, in the Alps and in the alpine parts of Northern Scandinavia (Figure S1). Projections of the species' climatic suitability into these regions must be interpreted with particular caution.

### Comparison of Interaction Approaches

All approaches showed high discriminatory model performance according to AUC, ranging from 0.88 to 0.94 (Table 1). Nevertheless, AUC values differ considerably between the approaches, especially between the ‘reference area approach’, which yielded the lowest AUC value of 0.88, and the others. The other approaches yielded higher and more similar values. Concerning omission and commission rates the ‘explanatory variable approach’ showed the lowest omission error, but the highest commission error compared to all other approaches (Table 1).

**Table 1:** Model performance and occurrence thresholds of the applied approaches.

Approach	AUC	Omission rate (%)	Commission rate (%)	Occurrence threshold
*Aeshna viridis* only	0.93	0.84	5.90	0.02
*Stratiotes aloides* only	0.94	6.05	5.81	0.35
Overlap	/	0.67	4.40	/
Explanatory variable	0.92	0.58	12.02	0.02
Reference area	0.88	1.20	1.88	0.05

As the ‘overlap approach’ represents the intersection of both species’ projected occurrences the AUC, threshold values, omission and commission rates of the single species modelling without interaction are shown.

The occurrence threshold is equivalent to the prevalence of the model-building data.

**Figure 4:**
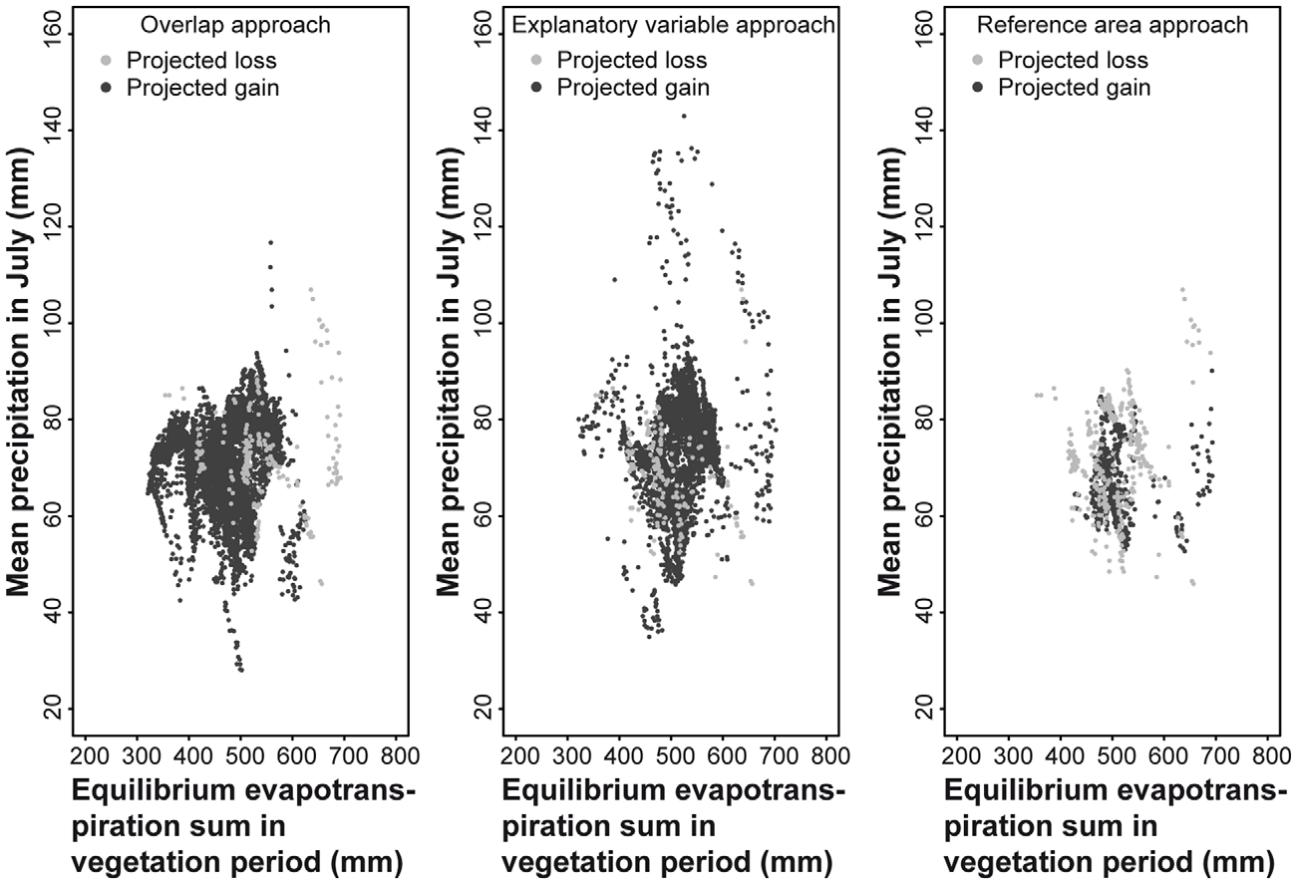
Projected future losses and gains of the current distribution of *Aeshna viridis* in Europe. Losses and gains are shown for the three applied biotic interaction approaches depending on the two most range-influencing climatic variables (out of six variables; variable importance measured by BIOMOD). Climate model: HadCM3, emission scenario: A2, time period: 2021–50. The vegetation period ranges from March until September.

Similarly, the differences in spatial patterns between the approaches are small but not negligible. The variable importance function in BIOMOD revealed the variables sum of equilibrium evapotranspiration in the vegetation period (March-September) and mean precipitation in July as the most important variables explaining the current distribution of *A. viridis* in Europe. For the ‘overlap approach’ most of the projected gaining points cover the range between 40 and 90 mm precipitation in July and 300 and 600 mm equilibrium evapotranspiration sum in the vegetation period (Figure 4). Losses are mainly projected between 600 and 700 mm equilibrium evapotranspiration sum in the vegetation period. The ‘explanatory variable approach’ shows a similar pattern. But in contrast to the ‘overlap approach’ additional gains are projected in the range of 600 and 700 mm evapotranspiration sum in the vegetation period and 90 till 140 mm precipitation in July. For the ‘reference area approach’ projected gains and losses cover similar ranges with precipitation in July mainly between 50 and 90 mm and equilibrium evapotranspiration sum in vegetation period between 450 and 550 mm representing a narrower range than the two other approaches. Compared to the current distribution (Figure S2) all biotic interaction approaches project gains in grid cells with climatic conditions that are currently not populated by *A. viridis*.

Regarding the contribution of algorithm and approach to the overall uncertainty, the main part of explained variation stems from the modelling algorithm (99.3%), and only a small part is attributed to the modelling approach (0.7%).

## Discussion

### Projected Changes in Spatial Distribution Patterns

The projected range changes for *A. viridis* up to 2021–50 were similar independent of the applied method for incorporating biotic interactions – namely ‘overlap’, ‘explanatory variable’ and ‘reference area approach’ – and of the modelling result for the target species *A. viridis* only. All model results projected a range expansion. However, smaller percentage gains were projected when biotic interactions were included. Similar results were found in a study where biotic variables were included in niche models for a butterfly and a bird species [44]. There, habitat availability was also reduced compared to a climate-only model although the species’ ranges generally declined. In our case, the essential egg-laying plant is projected to increase its range northwards, which could favour the spread of *A. viridis*.

The populations at the tail end of the distribution are regarded to be crucially important for the survival of a species due to high levels of regional genetic diversity and local adaptations [45]. The loss of genetic diversity, as expected through climate change, could mean the loss of potentially adaptive alleles leading to a lower adaptation potential and therefore to a higher extinction probability [46]. Here, *A. viridis* seems not to be affected by a loss of genetic diversity as range losses at the southern range margin are rarely projected. In this case, the projected range concentration around the Baltic Sea and its potential as leading edge for northward-directed range expansions might be a primary focus of nature conservation. However, a secondary focus on regions where a distribution loss might occur may be beneficial to maintain genetic diversity and local adaptation possibilities.

Another study showed that the incorporation of biotic interactions into species distribution modelling has an effect on the projections of the potential future distribution of a species [9]. They tested a modelling approach similar to our ‘explanatory variable approach’ with the result that the consideration of the host plant of *Parnassius mnemosyne* affected the projection of the species’ future potential distribution and significantly improved model performance. In our study, we could partly confirm this finding for a dragonfly and its specific egg-laying plant. The incorporation of the interaction affected the future spatial projections, although the performance criterion AUC did not improve. Other authors could also demonstrate an improvement of model performance with the inclusion of biotic interactions [47]. In addition, they suggest that species interactions may significantly affect distributions on macro-ecological scales at least for boreal birds.

Our expectation that a strong spatial mismatch between *A. viridis* and *S. aloides* might occur in the future is not supported by the modelling results. All applied modelling approaches resulted in remaining overlapping areas and showed similar tendencies in projected range losses and gains. Beside this spatial congruence a temporal mismatch could occur, which is not considered so far. Field studies could already prove temporal mismatches caused by climate change for different species with both positive (i.e. range expanding) (e.g. [10]) and negative (i.e. range declining) (e.g. [48]) effects on the studied populations. However, we suggest for our case that such a temporal mismatch is unlikely as *A. viridis* is not dependent on a specific stage of *S. aloides* (such as flowering), which is only available for a short time, but is rather dependent on the occurrence of the plant in general.

A host plant change as currently observed for the butterfly *Aricia agestis* in Great Britain and therewith a facilitation of range expansion [49] could be imaginable. However, a change of the egg-laying plant of *A. viridis* seems unlikely. Though *A. viridis* occasionally uses other plants, such as *Typha* spp. and *Sparganium* spp., only *S. aloides* provides shelter for the larvae against fish predation [14]. In a predation experiment they revealed a significant higher survival of larvae in tanks with *S. aloides* than in tanks without this plant.

### Interaction Approaches

The hypothesis that the three biotic interaction approaches differ considerably in their performance and their projected extent of range change is only partly supported by our results. The AUC values differed between the approaches to a varying extent, but all approaches exhibited high model performance. However, the value of the performance criterion did not improve with the inclusion of the host plant as additional predictive variable. Omission and commission rates were relatively small to moderate but nevertheless differed between approaches. The climate-only model of *A. viridis* yielded both a low omission and commission rate whereas the other approaches differed more in these rates. As an extreme example, the ‘explanatory variable approach’ had the lowest omission but the highest commission rate.

The projected geographical range changes were similar, concentrating the future potential suitable habitat around the Baltic Sea. All approaches projected range expansions in the north of the current distribution approving the recent findings of poleward range expansion of Odonata (e.g. [50], [51]). Additionally, the current distribution gaps of *A. viridis* in Central and Northern Europe could be closed provided that suitable habitat is available. Nevertheless, there were some geographical differences distinguishing the outcomes of the three biotic interaction approaches. The question is how important these differences are on the applied spatial scale. At a finer scale, other factors than climate, such as land use and habitat fragmentation, play a more important role for species performance [8] overruling the projected range changes and necessitating a more detailed look at the projected regions.

The projected losses and gains depending on the two most important variables and biotic interaction approach differ considerably. These differences may be caused by the different ways *S. aloides* affects the distribution of *A. viridis* in the approaches. Projected range gains in grid cells with currently unoccupied climatic conditions by *A. viridis* can be attributed to *S. aloides*. The egg-laying plant currently occurs in habitats with an equilibrium evaporation sum in the vegetation period up to approximately 800 mm and a mean precipitation in July between approximately 10 and 160 mm.

### Limitations

Absence data can be ambivalent, i.e. indicating unsuitable habitat or habitat that is suitable but unoccupied [5]. Further, for cryptic species or species that are difficult to detect in the field recorded absences might not be ‘real’ absences since the chance that the species occurs in a grid cell but is not detected is very high. Otherwise, presence-only data (such as museum data) often have strong sampling biases. Additionally, presence-only distribution modelling requires background (or pseudo-absence) data. The selection of such background data can influence model parameterization and therewith the accuracy of model projections [52]. Still, more detailed data is rarely available at continental scales.

Biotic interactions may play a minor role on a continental scale and climate seems to be the most important factor determining the distribution of species [8]. However, in Europe the spatial distribution of *A. viridis* is controlled by the occurrence of *S. aloides*, and is thus crucial at this spatial scale. In another study the incorporation of biotic interactions at macro-scales significantly improved projections of species distributions [47] and therewith partly disproved the minor importance of biotic interactions on larger macro-scales. Hence, it seems appropriate to include the biotic interaction between *A. viridis* and *S. aloides* in species distribution modelling even at a continental scale.

A study about uncertainty in the model-building process determined model algorithm and data quality as the most influential factors [53]. Similar to these results, here the main source of uncertainty is the modelling algorithm. We dealt with this uncertainty by using an ensemble modelling approach giving mean values of the projections over all modelling algorithms. The variation explained by the approach to incorporate biotic interactions is minimal, suggesting that the choice of a particular approach is not a significant source of prediction uncertainty. However, the incorporation of biotic interactions improves the model ability to explain the data variance.

The MESS analysis [42], comparing the novelty of climate between projected and calibrated space, revealed a large extent of non-analogue climate. While the ‘reference area’ approach is conceptually appealing, as it allows separating the effect of climate and occurrence of the host plant, the restriction of the model calibration area to the current occurrence of the host plant increases the extent of novel climate. The ensemble modelling and the threshold method for calculating presence-absence points from occurrence probabilities applied in this study reduced the effect of extreme projections. Nevertheless, the issue of non-analogue climate has to be kept in mind, especially when applying other modelling techniques that are more prone to make extreme predictions. We recommend a visualization of the different projections of the single algorithms to detect such projections into regions with non-analogue climate conditions.

All species distribution modelling approaches depend on the availability, quality and timeliness of distribution data [54]. The spatial resolution of distribution data provided by the EU 25 member states (report obligation of the Habitats Directive 2007) differs between countries. Non-EU countries, such as Switzerland, Norway, Ukraine or the Balkan States, are not listed in the Habitats Directive. Leaving occurrences in these countries out of consideration may distort the species distribution model. However, the availability of such data is often limited. European distribution data of plants, not listed in the Habitats Directive and not yet covered by the Atlas Florae Europaeae, can be most often only found in ‘old’ maps of distribution atlases, not necessarily representing the current distribution and mostly afflicted with sampling biases. The distribution data of *S. aloides* are from 1986 and may over- or underestimate the current distribution in Europe and therewith influence modelling results. Especially, the ‘reference area approach’ might be susceptible to incomplete occurrence data because of its model calibration on the range of the plant. Comparing the current distributions of both species *A. viridis* seems to occur where *S. aloides* does not exist. Two reasons for this are imaginable: Observed individuals of *A. viridis* are vagrants and do not breed there or the distribution map of *S. aloides* is incomplete at these places. Nevertheless, these databases provide a substantial and valuable source of distribution data in Europe.

Beside the well-studied uncertainties in forecasting species distribution modelling, such as the choice of model algorithm, climate model, emission scenario and so on, the selection of a certain threshold to convert occurrence probabilities into presence-absence points has remained a topic of debate. Several studies compared the performance of different thresholds (e.g. [36], [55], [56]) leading to different and even contrasting results in which threshold method performs best. We decided to use a threshold that equals the observed prevalence of the species in Europe. This has been shown to perform well with comparable high values for sensitivity, specificity and kappa [36]. However, this threshold resulted in low kappa values in another study [56]. Moreover, a recently published article documents that the choice of threshold is the second highest source of uncertainty following the modelling method [57]. Consequently, the choice of threshold can alter future range projections. In an extreme case, future projections may be reversed leading to projected range contractions (Figure S3) where with another threshold the range is projected to increase (Figure 3). Hence, it is important to evaluate the ecological plausibility of modelling results after deciding for a certain threshold.

All three here evaluated approaches for incorporating biotic interactions are static, i.e. they do not explicitly model range dynamics. Range dynamics of interacting species may lead to temporal mismatches, i.e. even if climatic conditions were suitable for both species, a lower range filling capacity of the host plant would limit the range expansion of the dependent species. Several approaches have been developed towards dynamic species distribution models, e.g. by coupling stochastic (meta-)population models with temporally varying species distribution models [58], [59] or dynamic range models [60]. To our knowledge, these approaches have not yet been expanded to take biotic interactions into account.

### Implications for future Modelling of Biotic Interactions

Many species, for example insect species of the EU Habitats Directive such as *A. viridis,* have highly specialised habitat requirements and fragmented distributions. Therefore, it is unlikely that they can colonise regions that become climatically favourable under climate change in the future. Hence, projections of the future distribution considering dispersal limitations and explicitly incorporating range dynamics may be more realistic for such species.

However, here we showed smaller range expansions to occur under a full dispersal scenario, only by including biotic interactions. Therefore, we conclude that for specialised species it is relevant to include biotic interactions in distribution modelling. Previous species distribution models without considering biotic interactions may have overestimated range gains and are over-optimistic in assessing future distributions.

## Supporting Information

Figure S1 **Results of the MESS-analysis for the ‘reference area approach’.** Light grey indicates a climatic similarity (values between 0 and 100) between calibrated (restricted to the current occurrence of *Stratiotes aloides*) and projected area (Europe). Dark grey areas (values <0) indicate novel climate conditions in the projected area.
(PDF)

Figure S2 **Distribution of** ***Aeshna viridis*** **depending on the two most range-influencing climatic variables.** The current distribution in Europe comprises 658 observed presence points. The vegetation period ranges from March until September.
(PDF)

Figure S3 **Projected potential future distributions of** ***Aeshna viridis*** **in Europe assuming unlimited dispersal.** The threshold for occurrence and non-occurrence projections was selected such that the resulting prevalence (i.e. fraction of occupied sites) equalled the mean predicted occurrence probability. A) *A. viridis* without interaction, occurrence threshold: 0.12, AUC: 0.93. B) Overlapping area of the potential future distributions of *A. viridis* and *S. aloides*, occurrence threshold: 0.12 (*A. viridis*), 0.44 (*S. aloides*), AUC: 0.93 (*A. viridis*), 0.94 (*S. aloides*). C) Considering the modelled occurrence probability of *S. aloides* in Europe as additional explanatory variable beside climate. Occurrence threshold: 0.10, AUC: 0.92. D) Potential future distribution of *A. viridis* in Europe applying the ‘reference area approach’. The model for *A. viridis* was calibrated within the distribution area of *S. aloides*. The modelled future occurrence probabilities of both species were multiplied. Occurrence threshold: 0.10, AUC: 0.88. All modelling results are based on an ensemble modelling with nine model algorithms with the climate model HadCM3 and the emission scenario A2 for the time period 2021–50.
(TIF)
