# Supplementary figures and images for: Biotic Interactions in the Face of Climate Change: A Comparison of Three Modelling Approaches
Source: PLoS One. 2012 Dec 6;7(12):e51472. doi: 10.1371/journal.pone.0051472 (PMC3516533; doi:10.1371/journal.pone.0051472)

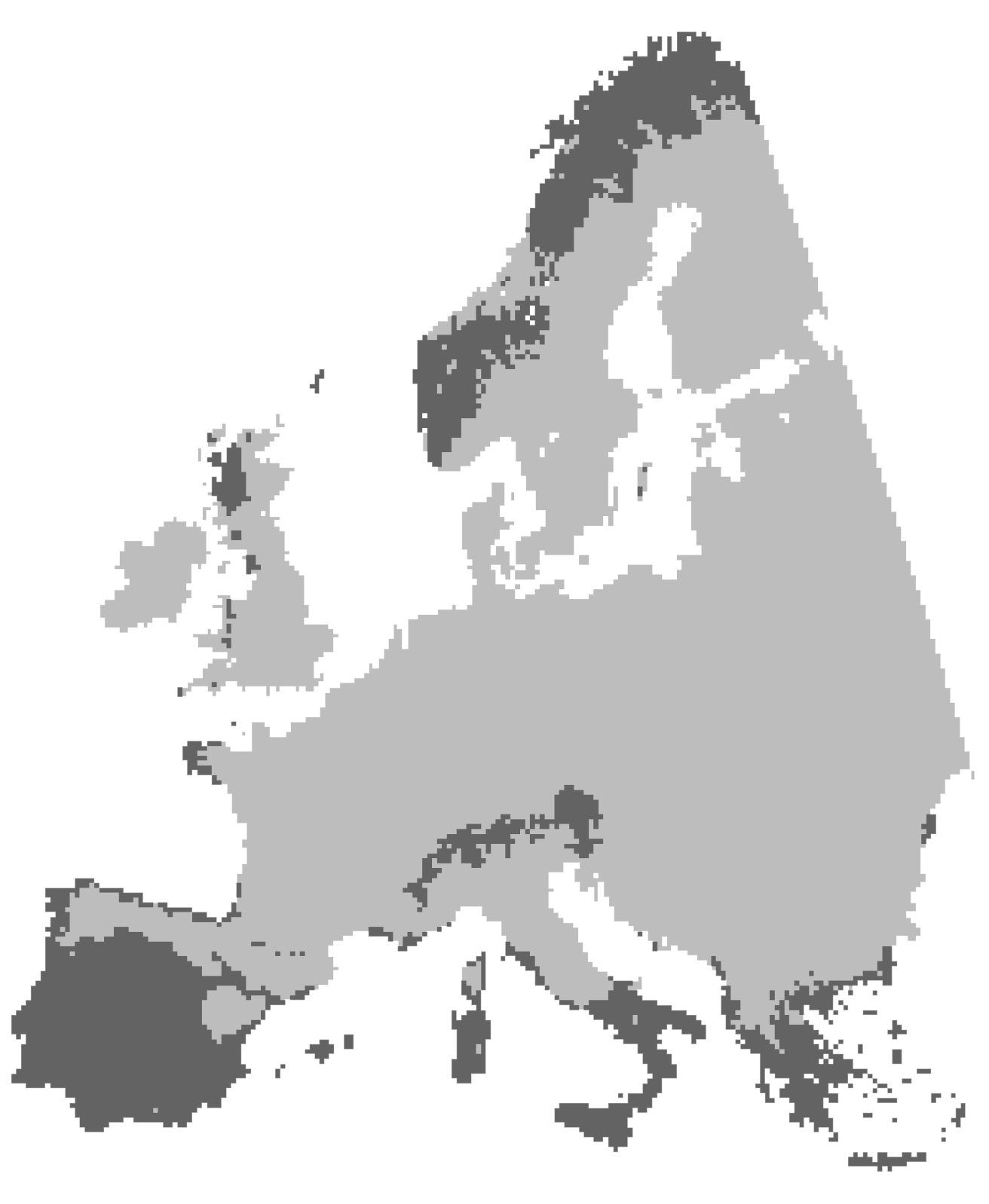

Supplement: Figure S1 — Results of the MESS-analysis for the ‘reference area approach’. Light grey indicates a climatic similarity (values between 0 and 100) between calibrated (restricted to the current occurrence of Stratiotes aloides) and projected area (Europe). Dark grey areas (values <0) indicate novel climate conditions in the projected area. (PDF) [file pone.0051472.s001.pdf]

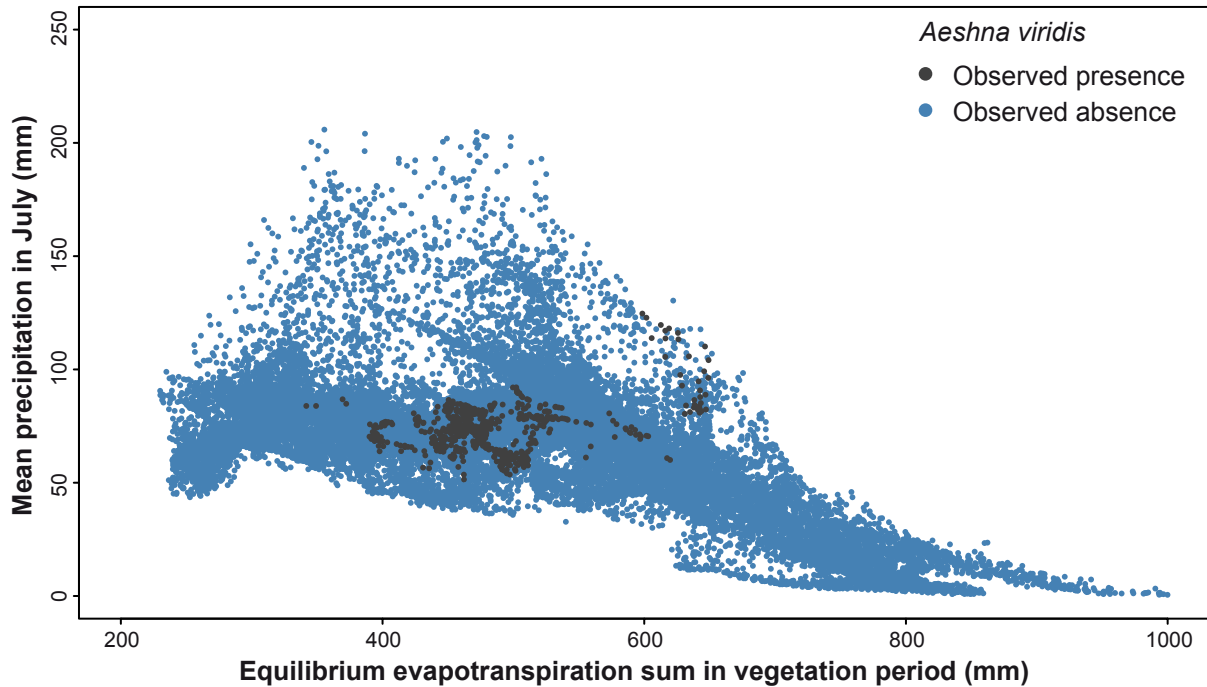

Supplement: Figure S2 — Distribution of Aeshna viridis depending on the two most range-influencing climatic variables. The current distribution in Europe comprises 658 observed presence points. The vegetation period ranges from March until September. (PDF) [file pone.0051472.s002.pdf]

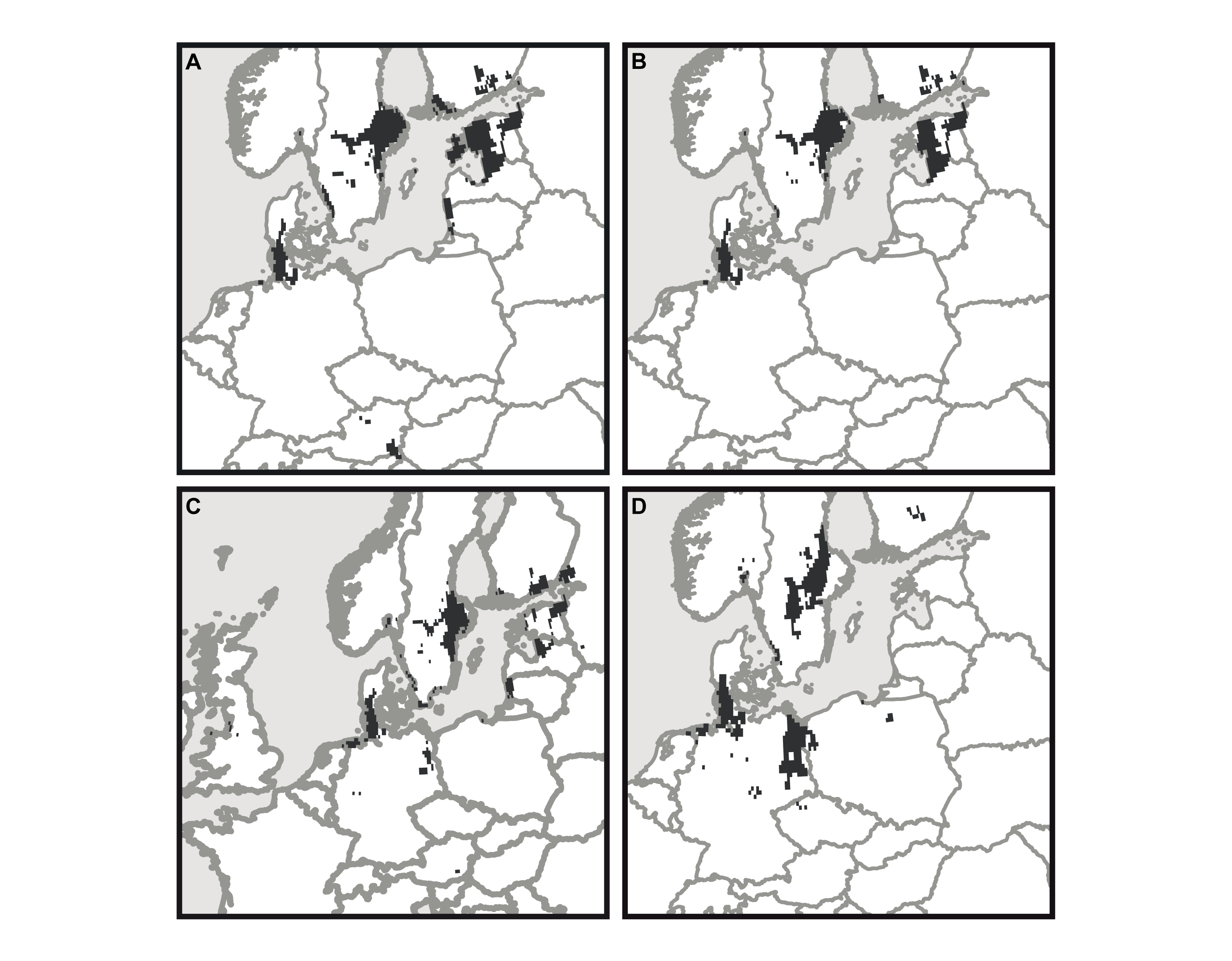

Supplement: Figure S3 — Projected potential future distributions of Aeshna viridis in Europe assuming unlimited dispersal. The threshold for occurrence and non-occurrence projections was selected such that the resulting prevalence (i.e. fraction of occupied sites) equalled the mean predicted occurrence probability. A) A. viridis without interaction, occurrence threshold: 0.12, AUC: 0.93. B) Overlapping area of the potential future distributions of A. viridis and S. aloides, occurrence threshold: 0.12 (A. viridis), 0.44 (S. aloides), AUC: 0.93 (A. viridis), 0.94 (S. aloides). C) Considering the modelled occurrence probability of S. aloides in Europe as additional explanatory variable beside climate. Occurrence threshold: 0.10, AUC: 0.92. D) Potential future distribution of A. viridis in Europe applying the ‘reference area approach’. The model for A. viridis was calibrated within the distribution area of S. aloides. The modelled future occurrence probabilities of both species were multiplied. Occurrence threshold: 0.10, AUC: 0.88. All modelling results are based on an ensemble modelling with nine model algorithms with the climate model HadCM3 and the emission scenario A2 for the time period 2021–50. (TIF) [file pone.0051472.s003.tif]
